# Neighbourhood-level income and Zika virus infection during pregnancy in Recife, Pernambuco, Brazil: an ecological perspective, 2015–2017

**DOI:** 10.1136/bmjgh-2021-006811

**Published:** 2021-12-02

**Authors:** Ludmila Lobkowicz, Grace M Power, Wayner Vieira De Souza, Ulisses Ramos Montarroyos, Celina Maria Turchi Martelli, Thalia Velho Barreto de Araùjo, Luciana Caroline Albuquerque Bezerra, Rafael Dhalia, Ernesto T A Marques, Demócrito de Barros Miranda-Filho, Elizabeth B Brickley, Ricardo Arraes de Alencar Ximenes

**Affiliations:** 1 Health Equity Action Lab, Department of Infectious Disease Epidemiology, London School of Hygiene & Tropical Medicine, London, UK; 2 Department of Disease Control, London School of Hygiene & Tropical Medicine, London, UK; 3 MRC Integrative Epidemiology Unit, Department of Population Health Sciences, Bristol Medical School, Bristol, UK; 4 Instituto Aggeu Magalhães, Fundação Oswaldo Cruz, Recife, PE, Brasil; 5 Instituto de Ciências Biológicas, Universidade de Pernambuco, Recife, PE, Brasil; 6 Departamento de Medicina Social, Universidade Federal de Pernambuco, Recife, PE, Brasil; 7 Secretaria Estadual de Saúde, Recife, PE, Brasil; 8 Department of Infectious Diseases and Microbiology, University of Pittsburgh, Pittsburgh, PA, USA; 9 Departamento de Medicina Interna, Universidade de Pernambuco, Recife, PE, Brasil; 10 Departamento de Medicina Tropical, Universidade Federal de Pernambuco, Recife, PE, Brasil

**Keywords:** arboviruses, epidemiology, infections, diseases, disorders, injuries, obstetrics

## Abstract

Zika virus (ZIKV) infections during pregnancy can lead to adverse neurodevelopmental and clinical outcomes in congenitally infected offspring. As the city of Recife in Pernambuco State, Brazil—the epicentre of the Brazilian microcephaly epidemic—has considerable disparities in living conditions, this study used an ecological approach to investigate the association between income at the neighbourhood level and the risk of ZIKV infections in pregnant individuals between December 2015 and April 2017. The spatial distribution of pregnant individuals with ZIKV infection was plotted on a map of Recife stratified into four categories based on mean monthly income of household heads. Additionally, a Poisson regression model with robust variance was fitted to compare proportions of ZIKV infections among pregnant individuals in relation to the mean monthly income of household heads, based on the 2010 census data, across 94 neighbourhoods in Recife. The results provide evidence that the risk of ZIKV infection to pregnant individuals was higher among those residing in lower-income neighbourhoods: relative to neighbourhoods that had a mean monthly income of ≥5 times minimum wage, neighbourhoods with <1 and 1 to <2 times minimum wage had more than four times the risk (incidence rate ratio, 95% CI 4.08, 1.88 to 8.85 and 4.30, 2.00 to 9.20, respectively). This study provides evidence of a strong association between neighbourhood-level income and ZIKV infection risks in the pregnant population of Recife. In settings prone to arboviral outbreaks, locally targeted interventions to improve living conditions, sanitation, and mosquito control should be a key focus of governmental interventions to reduce risks associated with ZIKV infections during pregnancy.

**Key questions**

**What is already known?**

- Zika virus (ZIKV) infections during pregnancy can lead to adverse neurodevelopmental and clinical outcomes in congenitally infected offspring.
- Risks of exposure to the *Aedes spp* mosquitoes that transmit ZIKV are strongly influenced by shared neighbourhood-level factors.
- Nevertheless, the association between neighbourhood-level income and ZIKV infection risks in the pregnant population remains uncertain.

**What are the new findings?**

- Between December 2015 and August 2017 in Recife, Pernambuco, Brazil, pregnant individuals residing in low-income and very low-income neighbourhoods experienced approximately four times the risk of acquiring ZIKV infections as compared with pregnant individuals residing in high-income neighbourhoods.

**What do the new findings imply?**

- These findings imply that neighbourhood-level income is a social determinant of ZIKV infections during pregnancy.
- In settings prone to arboviral outbreaks, locally targeted interventions to improve living conditions, sanitation and mosquito control should be a key focus of governmental interventions to reduce risks associated with ZIKV infections during pregnancy.

## Introduction

Zika virus (ZIKV) is an arthropod-borne virus (arbovirus), primarily transmitted to humans by the day-biting urban mosquito vector *Aedes aegypti*. Retrospective phylogenetic analyses suggest that the introduction of ZIKV into Brazil may have occurred as early as 2013, more than 1 year prior to its earliest clinical detection.^1^ Cases of acute exanthematous illness were increasingly reported towards the end of 2014 in several municipalities in Northeast Brazil^2^ and, by April 2015, ZIKV was recognised as the aetiological agent.^3^

Following the detection of clusters of newborns with microcephaly and other neurological impairments in the Brazilian regions most affected by the ZIKV epidemic, the Brazilian Ministry of Health declared a national emergency^4^, and the World Health Oragnization (WHO) designated the situation to be a Public Health Emergency of International Concern.^5^ Subsequent observational studies, including a case-control study reporting 73.1 (95% CI 13.0 to ∞) times higher odds of microcephaly among ZIKV-positive pregnancies,^6 7^ provided epidemiological evidence linking prenatal ZIKV infections and microcephaly. In parallel, an early case series of 104 congenitally infected children provided evidence that congenital Zika syndrome (CZS) could present with a wide spectrum of severity and a range of clinical features in addition to microcephaly, including facial disproportionality, cutis girata, hearing and ophthalmological abnormalities, hypertonia, spasticity, hyper-reflexia, irritability and abnormal neuroimaging (eg, calcifications, ventriculomegaly and lissencephaly).^8^

In total for the calendar year of 2016, the Brazilian National Notifiable Diseases Surveillance System (Sistema de Informação de Agravos de Notificação) reported 268 805 ZIKV cases, of which 24 143 (9.0%) were among pregnant individuals.^9^ A 2017 meta-analysis, summarising data from eight cohort studies across the Americas, estimated microcephaly prevalence to be 2.3% (95% CI 1.0% to 5.3%) among ZIKV-infected pregnancies^10^ and the prevalence of any adverse neurological findings to be as high as 5%–10%^10^; similar results were found in the prospective Microcephaly Epidemic Research Group (MERG) Pregnant Women’s Cohort in Pernambuco state, which estimated these figures to be 2.9% and 5.3%, respectively.^11^ Despite a global decline in transmission of ZIKV, new cases of children born with CZS have continued to be reported (eg, in Angola in 2018^12^ and in Lao People’s Democratic Republic in 2020),^13^ and the threat of a re-emergence of ZIKV persists. At the same time, thousands of families of children with CZS continue to grapple with significant health and social consequences from the last epidemic. Children with CZS have been observed to experience a vast range of clinical manifestations, including dysphagia,^14^ hearing and visual abnormalities,^15 16^ early epilepsy,^17^ neurodevelopmental delays,^18 19^ adenoid hypertrophy,^20^ cryptorchidism,^21 22^ endocrine dysfunction^23^ and an estimated case fatality rate of 10%.^24^

Previous studies examining arboviral infections and their sequelae (eg, for Dengue,^25–28^ Chikungunya^29 30^ and Zika^31^) have identified associations with poverty,^32 33^ examining specific risk factors including lower educational attainment,^34^ income,^26 30^ poor housing materials,^26 27^ race/racism^34 35^ and migration status.^28^ Mechanisms by which poverty may increase arboviral transmission may include individual-level factors (eg, lack of access to vector control interventions, such as unaffordable insecticides) as well as neighbourhood-level factors that can influence mosquito proliferation.^36^ In line with the recommendations of the WHO Commission on the Social Determinants of Health,^37^ a more thorough understanding of the political, social, and economic determinants of arboviral infections is required to develop effective interventions to mitigate the burden and consequences of ZIKV infections during pregnancy.^38^

To investigate the association between neighbourhood-level income and the risk of prenatal ZIKV infections, this study employs an ecological approach using spatial data on pregnant individuals with rash who were notified to the Center for Strategic Information on Health Surveillance in Pernambuco (*Centro de Informações Estratégicas de Vigilância em Saúde de Pernambuco*), Cievs/PE^39^ between December 2015 and April 2017 in the city of Recife, Pernambuco, and subsequently enrolled in the MERG Pregnant Women’s Cohort. Specifically, this analysis aims to: (1) plot the location of pregnant individuals with ZIKV on a map of the city of Recife, depicting neighbourhoods by income category and (2) investigate the association between the proportion of ZIKV infections among pregnant individuals and mean monthly income of household heads across neighbourhoods in Recife.

## Methods

### Setting and study design

This study was performed in the city of Recife, Pernambuco, Brazil.^39^ Recife has 1.6 million inhabitants within an area of approximately 219 km^2^, which is divided into 94 neighbourhoods based on operational units used in the demographic census.^1 40^ In 2010, Recife had a Gini coefficient of 0.69, indicating severe income inequality,^41^ and it is estimated that more than 30% of the population resides in households lacking municipal sewage.^6 39^ As risks of exposure to the *Aedes spp* mosquitoes that transmit ZIKV are strongly influenced by shared neighbourhood-level factors (eg, local housing quality), this study employed an ecological approach to assess the relationship between neighbourhood-level income groups and ZIKV infection risks among the pregnant population.

### Data sources

**Figure 1:**
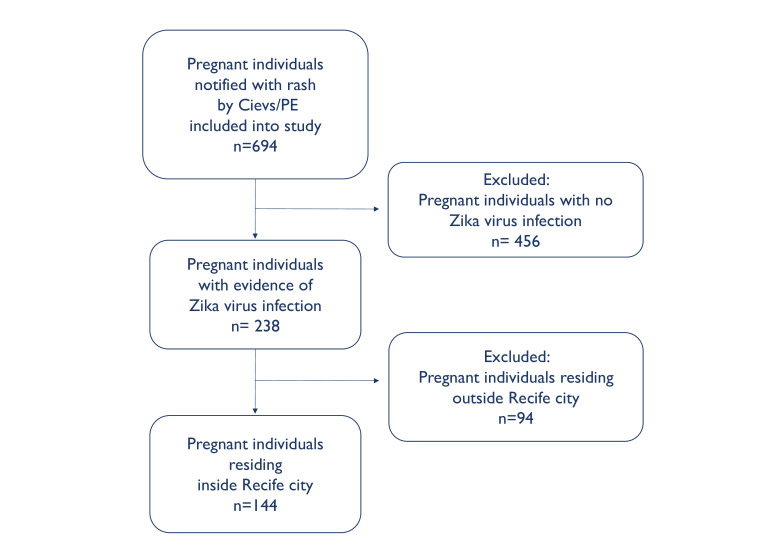
Flow diagram of study population in the Microcephaly Epidemic Research Group Pregnant Women’s Cohort. Cievs, *Centro de Informações Estratégicas de Vigilância em Saúde*; PE, Pernambuco.

This investigation brought together ecological data (ie, census tract-level information on mean household monthly income and number of children under the age of 1 year) from the 2010 Brazilian census (http://ghdx.healthdata.org/record/brazil-demographic-census-2010) and individual data from the prospective MERG cohort of pregnant individuals who presented with rash during the 2015–2017 ZIKV outbreak (for full study protocol, see: http://scf.cpqam.fiocruz.br/merg/).^39 42 43^ Although the 694 participants within the full MERG Pregnant Women’s Cohort included individuals residing within approximately 120 km of Recife, we restricted the current analysis to the city of Recife where the census data is most comparable with respect to urbanisation and environmental conditions (figure 1).

To determine a neighbourhood-level classification for income, data from the 2010 Brazilian census^42^ on the mean monthly income of household heads (hereafter, mean monthly income), 1843 census tracts were collapsed into the 94 neighbourhoods of Recife. This was achieved by computing the census tract population-weighted mean, for each neighbourhood, of the mean monthly income per tract. The dataset was then partitioned into four subgroups based on mean monthly income relative to minimum wage in Brazil in 2016 (ie, in 2016, R$880 per month)^44^: very low-income of <1 times minimum wage (<R$880 per month), low-income of 1 to <2 times minimum wage (R$880–R$1760 per month), medium-income of 2 to <5 times minimum wage (R$1760–R$4400 per month), and high-income of ≥5 times minimum wage (>R$4400 per month). These groupings ensured sufficient numbers within each category.

Between December 2015 and April 2017, pregnant individuals with rash (ie, a common sign of ZIKV infection) who were notified to Cievs/PE were invited to participate in the MERG Pregnant Women’s Cohort Study; no exclusion criteria were applied. Detailed information on the design of and ZIKV diagnostic testing in the cohort study has previously been described by Ximenes *et al*.^39^ Briefly, to identify ZIKV-infected pregnancies, sera were tested by a combination of quantitative reverse transcription PCR tests (qRT-PCR) using primers and probes described by Lanciotti *et al*,^45^ US Centers for Disease Control and Prevention capture immunoglobulin (Ig) IgM enzyme-linked immunosorbent assays (ELISAs), IgG3 ELISAs and/or Plaque Reduction Neutralisation Tests (PRNT_50_).^39^ As previously described, the longitudinal qRT-PCR and serological test results were considered in relation to the timing of rash onset during pregnancy and classified based on an evidence-graded diagnostic algorithm.^39^ Home addresses as well as data indicative of socioeconomic position of participants in the cohort were collected using a questionnaire administered at enrolment.

### Statistical analysis

To estimate the number of pregnancies occurring in each neighbourhood, this study assumed the number of children under 1 year as a proxy for the number of pregnant individuals in 2010. In order to compare the relative risks of maternal ZIKV infections between December 2015 and June 2017 at the neighbourhood-level within the four income groups, we estimated incident rate ratios, comparing the medium-income, low-income, and very low-income neighbourhoods relative to the high-income neighbourhoods in an unadjusted Poisson regression model with robust variance fitted using the proxy for pregnant individuals as the denominator. Data analysis was performed using Stata, V.13.0.^46^ To visualise the spatial distribution of maternal ZIKV infections in the city of Recife divided by living conditions, ArcGIS software^47^ was used to plot the residences of the ZIKV-infected pregnant individuals from the MERG cohort and layers indicating the four income strata of the 94 neighbourhoods onto a cartographic shapefile for the city of Recife (updated in 2010^42^) downloaded from the Brazilian Institute of Geography and Statistics (*Instituto Brasileiro de Geografia e Estatistica,* IBGE; https://mapas.ibge.gov.br/bases-e-referenciais/bases-cartograficas/malhas-digitais.html). The map was made at a scale of 1:100 000, which produces an error of approximately 20 m on the real scale, leading to each case being located in a broad circle of approximately 1250 m^2^.

**Patient and public involvement**

This research was done without patient or public involvement.

## Results

Between December 2015 and June 2017 in the city of Recife, 238 pregnant individuals with rash were notified to the Cievs/PE surveillance system and consented to participate in the MERG Pregnant Women’s Cohort. Of these, 144 were confirmed to have laboratory evidence of ZIKV infection during pregnancy (table 1, figure 1). Among the 144 confirmed cases, 71.5% self-identified as mixed-race (*Parda*), 21.5% as White Brazilians (*Branca*), 6.3% as Black Afro-Brazilians (*Preta*) and 0.7% as East Asian Brazilians (*Amarela*). The majority of cases (n=133, 92.4%), had a primary school education, whereas only 11 (7.6%) reported secondary or higher education. Fewer than half of the cases (n=67, 46.5%), reported household ownership of a computer.

**Table 1:** Baseline characteristics among Zika virus infected pregnant individuals with rash in the city of Recife notified to the Center for Strategic Information on Health Surveillance in Pernambuco (*Centro de Informações Estratégicas de Vigilância em Saúde de Pernambuco*) surveillance system from December 2015 to June 2017 (n=144 pregnant individuals)

	Category	No (%)
Race/ethnicity	Mixed-race (*Parda*)	103 (71.5)
	White Brazilian (*Branca*)	31 (21.5)
	Black Afro-Brazilian (*Preta*)	9 (6.3)
	East Asian-Brazilian (*Amarela*)	1 (0.7)
Educational attainment	Primary education, partial or completed	133 (92.4)
	Secondary education, partial or completed	6 (4.2)
	Higher education, partial or completed	5 (3.5)
Household crowding index (individuals in the house/rooms in the house)	>1.25	2 (1.4)
	1.00	33 (22.9)
	0.75	39 (27.1)
	0.50	62 (43.1)
	<0.25	8 (5.6)
Household asset ownership (at least one)	Bathroom	140 (97.2)
	Refrigerator	139 (96.5)
	Digital video disc player	101 (70.1)
	Microwave oven	85 (59.0)
	Laptop or desktop computer	67 (46.5)
	Washing machine	59 (41.0)
	Freezer	55 (38.2)
	Motorcycle	25 (17.4)
	Vehicle	3 (2.1)
	Dishwasher	2 (1.4)
	Dryer	1 (0.7)

**Figure 2:**
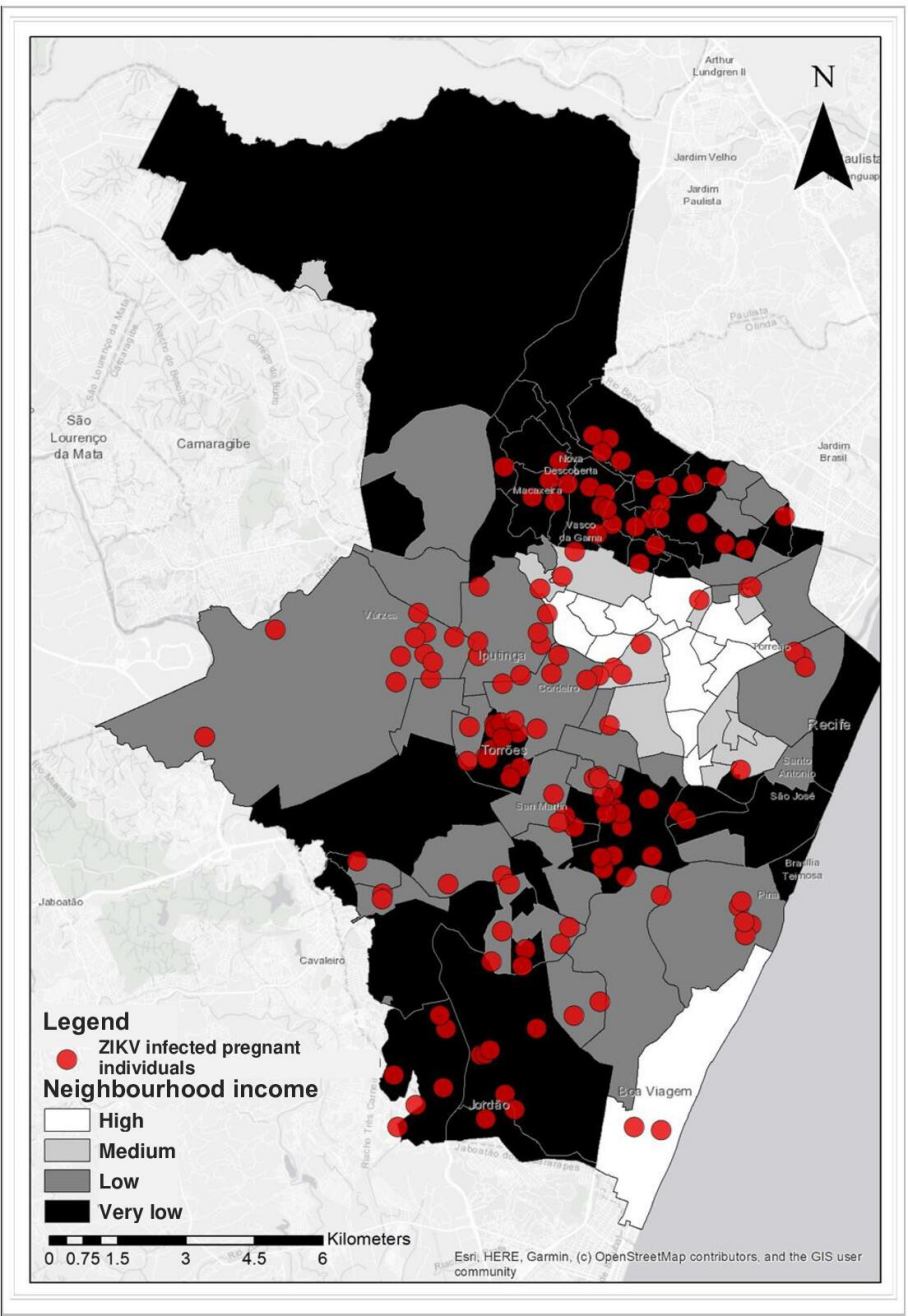
Zika virus (ZIKV) infected pregnant individuals (red dots) plotted on a map of the city of Recife, that is, depicting the 94 neighbourhoods of the city divided into areas of very low income (<1× minimum wage), low income (1 to <2× minimum wage) and medium income (2 to <5× minimum wage) compared with high income (≥5 × minimum wage). The scale of the map is 1:100 000.

During the study period, maternal ZIKV infections were detected in 36 of the 94 neighbourhoods in Recife, including 23 of the 37 (62.1%) very low-income neighbourhoods, 4 of the 32 (12.5%) low-income neighbourhoods, 3 of the 12 (25.0%) medium-income neighbourhoods and 4 of the 13 (30.8%) high-income neighbourhoods (figure 2). More than half of the maternal ZIKV infections (n=75, 52.1%) occurred in the 37 neighbourhoods that were in the very low-income bracket (table 2), while fewer than 3% of infections (N=4) were identified in the 13 high-income neighbourhoods. The incidence rate ratio of maternal infections during December 2015 and April 2017 in the very low-income, low-income and medium-income groups were 4.08 (95% CI 1.88 to 8.85), 4.30 (95% CI 2.00 to 9.20) and 3.11 (95% CI 1.25 to 7.76) times higher than those of the high-income group that had a mean monthly income at the neighbourhood level of ≥5 times minimum wage (table 2).

**Table 2:** Incidence rate ratio (IRR) of Zka virus infected pregnant individuals living in neighbourhoods in the city of Recife grouped by mean monthly income of household heads

Mean monthly income of household heads	No of neighbourhoods per group	Mean monthly income (R$)	No (row %) of pregnant individuals with ZIKV	No of children under 1 year	IRR	95% CI
Highest income (≥5 × minimum wage)	13	5098	4 (0.04%)	9298	1.00	Ref
Medium income (2 to <5 × minimum wage)	12	3024	7 (0.10%)	6829	3.11	1.25 to 7.76
Low income (1 to <2 × minimum wage)	32	1281	58 (5.10%)	1138	4.30	2.00 to 9.20
Very low income (<1 × minimum wage)	37	644	75 (3.71%)	2023	4.08	1.88 to 8.85

BRL, Brazilian Real.

## Discussion

The findings of this study provide evidence of an increased risk of ZIKV infections during pregnancy in relatively lower income communities in Brazil. This study illustrated that in neighbourhoods of Recife, Pernambuco, Brazil, where the mean monthly income of household heads was less than two times the minimum wage, pregnant individuals experienced approximately four times the risk of ZIKV infections between December 2015 and August 2017 compared with those in neighbourhoods where the mean monthly income was at least five times higher than the minimum wage.

These results are consistent with previous observational studies from Northeast Brazil that have provided evidence of an association between poverty indicators and arboviral infections. As described by Souza *et al* in their 2018 ecological investigation on living conditions and microcephaly incidence, Recife’s temperature and humidity levels combined with clusters of inadequate housing in overcrowded areas with minimal basic sanitation provide an ideal setting for the transmission of vector-borne diseases.^48^ They found that those residing in areas that had a low mean monthly income, which was used as a proxy for precarious living conditions, had a higher prevalence of microcephaly associated with ZIKV compared with those living in areas with higher mean incomes.^48^ Furthermore, a cross-sectional study conducted between 2005 and 2006 also investigated risk factors for dengue virus infection in socioeconomically distinct areas in Recife.^25^ Dengue seroprevalence was 91.1% in the socioeconomic area classified as deprived, 87.4% in that classified as intermediate and 74.3% in that classified as high. In addition, between 2015 and 2016 another seroprevalence survey was performed in Salvador, the capital of the neighbouring state of Bahia, and reported the infection rate of ZIKV to be higher in areas with high socioeconomic disadvantage.^49^

This study is an important analysis, investigating the association between ZIKV infection and income at the neighbourhood level in Recife, Pernambuco—the epicentre of the Brazilian microcephaly epidemic. Other strengths include the stringent testing for ZIKV infections during pregnancy, which was confirmed by testing serial blood samples with a combination of molecular and serological assays.^39^ In addition, a rigorous surveillance system initiated by the Pernambuco State Health Department in collaboration with MERG,^39^ which investigated all pregnant individuals presenting with rash, was used to identify and recruit participants. A potential limitation of this study is the use of a proxy for the number of pregnant individuals in each neighbourhood. The total number of children under the age of 1 year per neighbourhood in 2010, however, while not exact, is an informative reflection of the total number of pregnant individuals per neighbourhood. Importantly, population-based data from the 2006 National Demographic and Health of Children and Women from the Ministry of Health of Brazil reports 8.9% miscarriages and 1.5% induced abortions among the total annual number of pregnancies in Brazil, which have not been included in the proxy.^50^ Furthermore, since our mean monthly income estimates are based on the Brazilian census from 2010, the last Brazilian census conducted, there may have been some changes to income distribution in these neighbourhoods, resulting in potential inaccuracies. There is also potential for misclassification in the neighbourhood-level income variable. Specifically, there may be the risk of responder bias in household head recall of income. In addition, since neighbourhoods are not homogeneous in relation to socioeconomic conditions, the association between localised income and ZIKV infection risks may indeed be stronger than our results indicate. Further research to determine whether the findings of this study are consistent across the household and individual level would be complementary to this body of work and would further inform the use of public health interventions targeting households (eg, indoor residual spraying) and individuals (eg, repellent lotions). Additionally, as non-white individuals are disproportionately represented within the ZIKV-exposed population of this study as compared with the general population of Recife, an intersectional analysis considering other structural determinants, such as racial discrimination, would further improve our understanding of the high-risk groups for targeted public health intervention.

## Conclusions

The increasing frequency of arboviral epidemics is a growing public health concern. Contributing to our understanding of health inequity in the context of Brazil, our findings provide evidence of an increased incidence of ZIKV infections in urban areas populated by those with low incomes compared with those residing in areas of middle and higher incomes. Addressing underlying income poverty-associated environmental determinants, such as living conditions, sanitation, and mosquito control, should be a key focus of governmental interventions to reduce arboviral transmission in settings prone to outbreaks, such as Northeast Brazil. Wider interventions to reduce poverty may have ancillary benefits for the control of vector-borne diseases.

## Data Availability

Data are available on reasonable request. Data cannot be shared publicly because public availability would compromise patient privacy. Deidentified data can be made available on reasonable request from qualified investigators.
